# Depletion of histone demethylase KDM5B inhibits cell proliferation of hepatocellular carcinoma by regulation of cell cycle checkpoint proteins p15 and p27

**DOI:** 10.1186/s13046-016-0311-5

**Published:** 2016-02-25

**Authors:** Dong Wang, Sheng Han, Rui Peng, Chenyu Jiao, Xing Wang, Xinxiang Yang, Renjie Yang, Xiangcheng Li

**Affiliations:** Liver Transplantation Center, Key Laboratory of Living Donor Liver Transplantation, Ministry of Public Health, First Affiliated Hospital of Nanjing Medical University, 300 Guangzhou Road, Nanjing, 210029 China; Department of General Surgery, Nanjing Medical University Affiliated Cancer Hospital, Cancer Institute of Jiangsu Province, Nanjing, China

**Keywords:** KDM5B, HCC, Cell cycle, p15, p27

## Abstract

**Background:**

KDM5B is a jmjc domain-containing histone demethylase which remove tri-, di-, and monomethyl groups from histone H3 lysine 4 (H3K4). KDM5B has been determined as an oncogene in many malignancies. However, its expression and role in hepatocellular carcinoma (HCC) remain unknown.

**Methods:**

We detected the expression of KDM5B in HCC tissues and cell lines. Cell proliferation was performed to reveal the role of KDM5B depletion on HCC cells both in vivo and in vitro. Flow cytometry was used to analyze the cell cycle and chip analysis was conducted to determine the direct target of KDM5B.

**Results:**

KDM5B is frequently up-regulated in HCC specimens compared with adjacent normal tissues and its expression level was significantly correlated with tumor size, TNM stage, and Edmondson grade. Moreover, Kaplan-Meier survival analysis showed that patients with high levels of KDM5B expression had a relatively poor prognosis. Knockdown of KDM5B notably inhibits HCC cell proliferation both in vivo and in vitro via arresting the cell cycle at G1/S phase partly through up-regulation of p15 and p27. Further molecular mechanism study indicates that silencing of KDM5B promotes p15 and p27 expression by increasing histone H3K4 trimethylation in their promoters.

**Conclusions:**

KDM5B could be a potentially therapeutic target, which provides a rationale for the development of histone demethylase inhibitors as a strategy against HCC.

**Electronic supplementary material:**

The online version of this article (doi:10.1186/s13046-016-0311-5) contains supplementary material, which is available to authorized users.

## Background

Mounting evidence indicates that epigenetic alterations contribute significantly to the initiation and progression of multiple human malignancies, including hepatocellular carcinoma [1–3]. As an important type of histone modification, Histone methylation plays a central role in regulating chromatin dynamics and transcription. This modification has been linked to transcriptional activation or repression of gene expression, depending on the specific residues that become methylated and the state of methylation [4]. In general, lysine methylation on H3K4, H3K36, H3K79 is associated with gene activation, whereas lysine methylation on H3K9, H3K27, H4K20 is linked to gene silencing [5]. Histone lysine methylation is reversibly controlled by histone demethylases and methyltransferases. Growing studies suggest that a number of histone demethylases are dysregulated in tumors and can serve as oncoproteins [6–9].

Liver cancer is the sixth most common malignant disease worldwide but the third most frequent cause of cancer-related death, with only 5 % of patients surviving more than 5 years. In fact, about half of these cases and deaths occur in China. Hepatocellular carcinoma (HCC) represents the major histological subtype of primary liver cancer, accounting for 70 to 85 % of the total liver cancer worldwide, and its molecular etiology is heterogeneous [10]. Numerous studies of molecules and signalling pathways related to the development of HCC have been identified, yet the deep mechanisms underlying the oncogenesis and cancer progression of HCC remain poorly understood [11–15]. Therefore, a histone demethylase that promotes HCC tumorigenesis could be an attractive novel target for drug discovery. However, little is known about the role of histone demethylases in HCC.

KDM5B is a jmjc domain-containing histone demethylase, which belongs to KDM5 family. KDM5B was first identified and characterized in 1999 when Lu found its overexpression in human breast-cancer cell lines and primary breast carcinomas [16]. Mammalian KDM5B shows a restricted expression pattern in normal adult tissues and is primarily present in the testis and brain [17, 18]. However, KDM5B levels were found to be up-regulated in a variety of human cancers such as bladder cancer, [19] lung cancer, [20] colorectal cancer, [21] prostate cancer, [22, 23] gastric cancer, [24] glioma, [25] ovarian cancer [26] and malignant melanoma [27]. Since KDM5B probably acts as a transcriptional regulator by its ability to remove tri-, di-, and monomethyl groups from H3K4, several cancer-associated genes regulated by KDM5B have been identified, including tumor suppressor gene BRCA1 that is repressed by KDM5B and transcription factors E2F1 and E2F2 that are up-regulated by KDM5B [19, 28, 29]. Besides, increasing studies revealed that KDM5B was involved not only in tumor initiation, but also in tumor progression such as invasion and metastasis [30, 31]. Considering the growing evidence for an important function of KDM5B proteins in cancer, it becomes an attractive target for chemotherapeutic drug design. Several preclinical studies suggest inhibition of KDM5B histone demethylase can suppress tumorigenesis and provide strong rationale for development of their inhibitors for use in cancer therapy [32–34].

We are particularly interested in KDM5B because we found that it was the most significantly up-regulated histone demethylase among 27 histone demethylase family members by comparing their expression profiles between HCC and their adjacent normal liver tissues using public database. Then we determined whether the highly expressed KDM5B could contribute to the pathogenesis of HCC. In the present work, we demonstrate that KDM5B is frequently up-regulated in HCC specimens and cell lines. Knockdown of KDM5B significantly inhibits cellular proliferation of HCC cells and arrests cell cycle progression at the G1/S-phase. Moreover, the effect of KDM5B knockdown on HCC cell proliferation is mediated through upregulation of the cyclin-dependent kinase inhibitors, p15 and p27. Furthermore, our molecular mechanism study indicates that silencing of KDM5B promotes p15 and p27 expression by increasing histone H3K4 trimethylation in their promoters. Collectively, our findings provide new insight into the molecular pathogenesis of HCC and KDM5B may constitute a promising novel therapeutic target for blocking progression of HCC.

## Methods

### Tissue specimens

All HCC specimens were obtained from HCC patients who underwent surgical resection with informed consent from May 2008 to October 2011. Both the tumor and adjacent normal tissue were collected from each patient and were frozen at −80 °C until processed. All patients had negative histories of exposure to either chemotherapy or radiotherapy prior to the surgical operation. The diagnosis of HCC was validated by two individual pathologists. The use of human tissues was approved by the institutional ethics committee of Nanjing Medical University.

### Cell culture and culture conditions

The human HCC cell lines (Hep-3B, MHCC-97 L, SMMC-7721, SK-hep-1, MHCC-LM3, PLC/PRF/5, MHCC-LM6, YY-8103, FOCUS, MHCC-97H, HepG2, Huh7, WRL-68, Bel-7404, Bel-7405, L02) were obtained from the Chinese National Human Genome Center at Shanghai. Cells were grown in Dulbecco’s minimal essential medium (DMEM) supplemented with 10 % FBS (Gibco, USA) and antibiotics (50 U/ml penicillin and 50 μg/ml streptomycin) at an atmosphere of 5 % CO2.

### RNA extraction and quantitative real-time PCR

Total RNA was extracted from tissue or cell culture samples using TRIZOL reagent (Invitrogen) following the manufacturer’s protocol and was reverse transcribed into cDNA with a M-MLV reverse transcriptase kit (Promega, USA). Quantitative real-time PCR was performed using the Thermal Cycler Dice Detection System with the SYBR Premix Ex Taq™ (Takara, Japan). All samples were done in triplicate and the housekeeping gene β-actin was used as an endogenous control. The following primers were used to specially amplify the KDM5B gene and β-actin. For KDM5B, forward: 5-GGTGAGCCAAAAACCTGGTA-3, and reverse, 5-AATCACAAACTCCCCAGCAC-3; For β-actin: forward: 5-AGAGCCTCGCCTTTGCCGATCC-3, and reverse, 5-CTGGGCCTCGTCGCCCACATA-3.

### RNA interference and cell transfection

KDM5B-specific small interference RNAs (siRNAs) were designed and chemically synthesized (GenePharma, China) to suppress endogenous KDM5B expression in HCC cells: si-370, 5-CACGUAUCCAGAGACUGAAUGAAdTdT-3; si-2531, 5-GGAUGCAGAGAAGUGUGCCUCUGdTdT-3.

In addition, si-NC was also synthesized to act as a negative control: si-NC, 5-UUCUCCGAACGUGUCACGUdTdT-3.

The oligonucleotides encoding short hairpin RNAs (shRNAs) for the continuous knockdown of endogenous KDM5B were synthezised and inserted into pSUPER (Oligoengine, USA): sh-370, 5-GATCCCCCACGTATCCAGAGACTGAATGAATTCAAGAGAAATTCATTCAGTCTCTGGATACGTGTTTTTGGAAA-3; sh-2531, 5-GATCCCCGGATGCAGAGAAGTGTGCCTCTGTTCAAGAGACAGAGGCACACTTCTGCATCCTTTTTGGAAA-3; sh-NC containing irrelevant nucleotides was served as a negative control: sh-NC, 5-GATCCCCTTCTCCGAACGTGTCACGTTTCAAGAGAACGTGACACGTTCGGAGAATTTTTGGAAA-3. All constructs were fully sequenced. The cells were then transfected with siRNA or shRNA using lipofectamine 2000 (Invitrogen, USA) according to the manufacturer’s instructions at cell density of 30-50 %.

### Cell proliferation

Transiently transfected HCC cells were seeded at a density of 2000–5000 cells/well into 96-well plates and cultured for a week. Cell growth was examined by Cell Counting Kit-8 (Dojindo, Japan) according to the manufacturer’s instructions. The absorbance value at a wave length of 450 nm was used as an indicator of cell viability.

### Colony formation

Transfected HCC cells were plated at a density of 10,000–50,000 cells/10-cm plate and maintained in the medium with addition of 0.8 mg/ml G418 (Life Technologies, USA) for colony formation. After 3 weeks, colonies stained with 0.1 % crystal violet were counted and photographed.

For the soft agar colony formation assay, 2000–5000 transfected cells were plated and grown in the 24-well plate with the medium containing 1 % base agar and 0.5 % top agar. After 3 weeks, all colonies were counted and photographed under a dissecting microscope.

### Experiment in vivo

Animal care and euthanasia were approved by the Nanjing medical University animal studies committee. Fifteen BALB/c nude mice were randomly divided into three groups including the negative control group and two KDM5B knockdown groups. Hep3B cells transfected with sh-KDM5B or negative control were selected under 0.8 mg/ml G418 (Life Technologies, USA) for 3 weeks. Then the stably transfected cells were harvested at a concentration of 1 × 107 cells/ml. Of the suspending cells, 0.1 ml was inoculated subcutaneously into either side of the posterior flank of the 4–6-week-old BALB/C nude mice. Xenografts in each group were observed periodically after injection. Nude mice were killed by cervical dislocation after 3 weeks and the xenografts were peeled off subcutaneously. The weight of the xenografts in each group was compared and used for further analysis.

### Cell cycle analysis

Flow cytometry was performed to analyze the cell cycle. Serum starvation was used to induce cell cycle synchronization before cell transfection. At 48 h after transfection with KDM5B siRNAs duplexes, cells were harvested as single cell suspensions. After fixation in 70 % ethanol for 1 h at −20 °C, cells were washed and resuspended in PBS, followed by incubation with propidium iodide (10 mg/ml) and RNase A (10 mg/ml) for 30 min at 4 °C. FACSCalibur fow cytometer, CellQuest (BD Biosciences, USA) was used to calculate the percentage of cells in each phase.

### Protein extraction and western blot analysis

Total protein was extracted using cell lysis buffer containing 50 mM Tris–HCl (pH 6.8), 2 % SDS, 10 % 2-mercaptoethanol, 10 % glycerol, and protease inhibitor cocktail (Sigma, USA). Protein samples (20 mg) were resolved using SDS-PAGE and transferred onto a nitrocellulose membrane. The membrane was blocked with 5 % milk in TBS for 2 h at room temperature. After incubation with the appropriate primary antibody overnight at 4 °C, membranes were washed and incubated with the IRDye 800cW or 680RD secondary antibodies in TBST for 2 h at room temperature. Then the protein bands of interest were visualized with the Odyssey system (LI-COR, USA). The specific antibodies used in our study were as follows: anti-KDM5B (Abcam, British), anti-p15 (CST, USA), anti-p27 (CST, USA), anti-H3K4me2 (CST, USA), anti-H3K4me3 (CST, USA), anti-total-H3 (CST, USA), and anti-β-actin (CST, USA).

### Chromatin immunoprecipitation assays (ChIP)

The ChIP assays were performed by using EZ-ChIP KIT according to the manufacturer’s instruction (Millipore, USA). The control and KDM5B siRNA-treated Hep3B and Focus cells were treated with formaldehyde and incubated for 10 min to generate DNA-protein cross-links. Then the cell lysates were sonicated to generate chromatin fragments of 200–1000 bp. The antibodies against KDM5B and trimethylated H3-K4 (H3-K4me3) were used to precipitate DNA fragments bound by their corresponding elements. The protein-DNA complex was collected with protein A Sepharose beads (Millipore, USA), eluted, and reverse cross-linked. Following treatment with Protease K (Sigma-Aldrich, USA), the samples were extracted with phenol-chloroform and precipitated with ethanol. The recovered DNA was resuspended in TE buffer and used for the PCR amplification.

### Statistical analysis

All statistical data were carried out using Statistical Program for Social Sciences 18.0 software (SPSS, USA) and presented with Graphpad prism 5.0 (GraphPad Software, CA). The significance of differences between groups was evaluated with Student’s t-test and χ2 test as appropriate. DFS and OS rates were calculated by the Kaplan-Meier method with the log-rank test applied for comparison. Two-sided *p*-values were calculated, and a probability level of less than 0.05 was considered to be statistically significant.

## Results

### KDM5B is frequently up-regulated in HCC tissues and cell lines

**Fig. 1:**
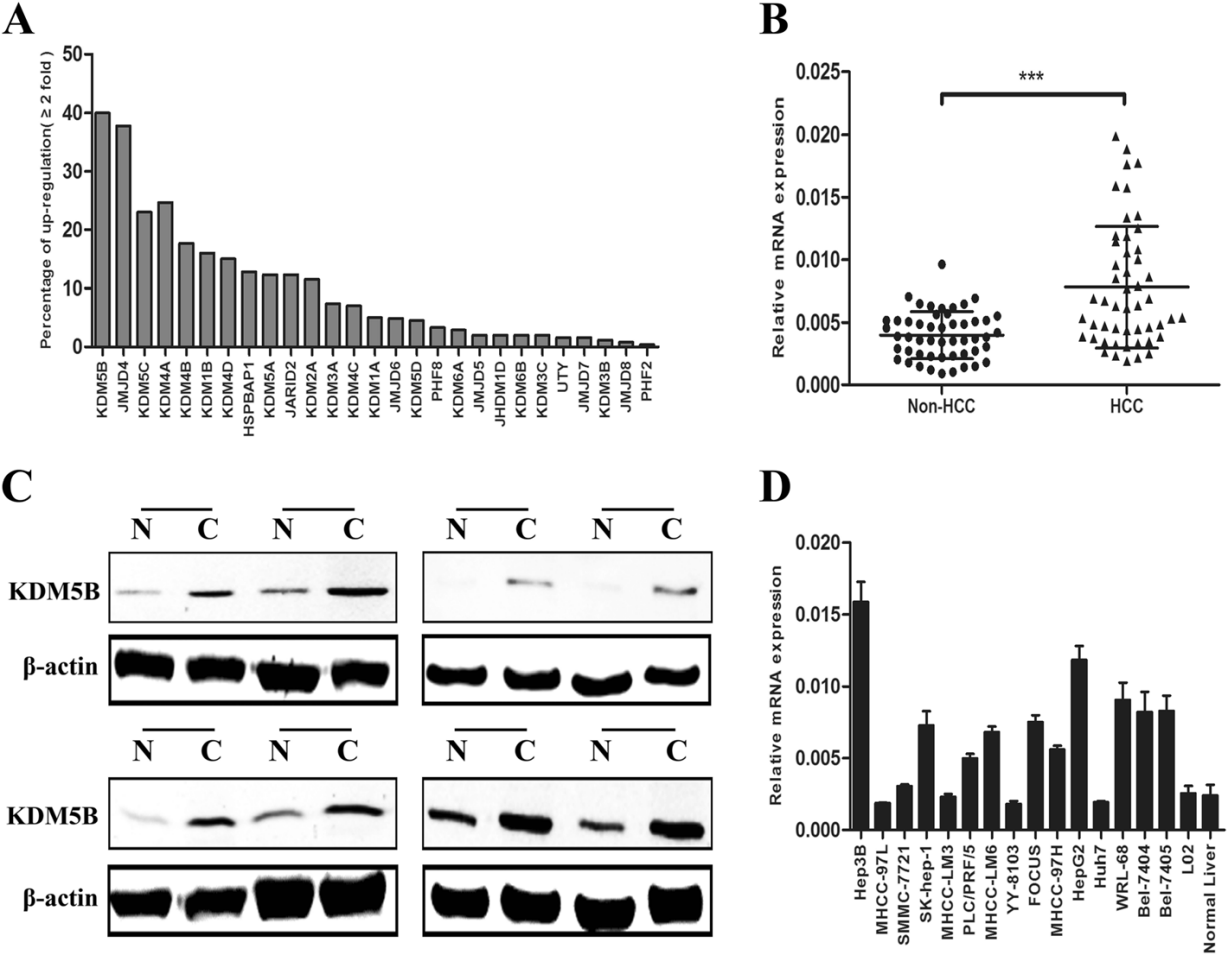
KDM5B expression is obviously up-regulated in HCC tissues and cell lines. **a** Expression profiles of a number of histone demethylase genes in a subset of HCC samples from public GEO Profiles database. **b** Validation of the aberrantly up-regulated KDM5B in 50 pairs of HCC and the corresponding adjacent non-cancerous liver (non-HCC) by quantitative real-time PCR. Expression of β-actin was used as an internal control and *P* value was calculated by student’s t test. (***, *P* < 0.001). **c** Representative results of up-regulation of KDM5B in 8 pairs of HCC (**c**) and the adjacent non-HCC liver tissue (N) by western blot. **d** The expression levels of KDM5B was determined by real-time PCR in 15 HCC cell lines as well as the normal human liver cell line L02 and normal adult liver tissue, with β-actin as an internal control

To explore a histone demethylase with oncogenic characteristics for HCC, We first analyzed expression profiles of a number of histone demethylase genes in a subset of HCC samples by using public GEO Profiles database (GSE 25097) and found that KDM5B was the most obvious up-regulated histone demethylase with at least 2-fold up-regulation in 40 % HCC specimens (Fig. 1a). To verify the data from public database, we used quantitative RT-PCR to measure KDM5B mRNA levels of 50 paired HCC samples and found significantly higher expression levels of KDM5B in HCC tissues compared with their corresponding non-tumorous liver tissue (Fig. 1b). Eight pairs of typical cases were illustrated by western blot (Fig. 1c). Subsequently, we analysed the expression profiles of 15 widely used HCC cell lines and the resulting data showed that the expression of KDM5B was elevated in most of HCC cell lines when compared to the normal human liver cell line L02 and normal adult liver tissue (Fig. 1d). Collectively, these results suggested that KDM5B expression is up-regulated in human HCC tissues and cell lines.

### KDM5B mRNA level is associated with clinicopathologic characteristics of HCC and high KDM5B expression predicts poor survival in HCC patients

**Fig. 2:**
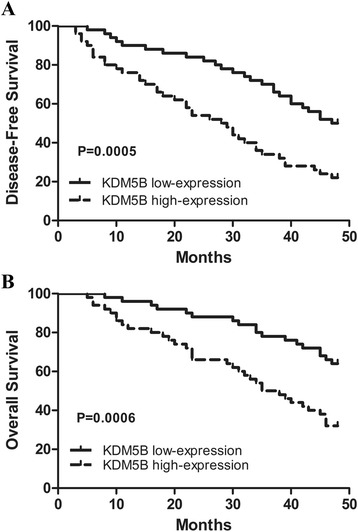
KDM5B expression was correlated with the DFS or OS of HCC patients. **a** Patients with high KDM5B expression had a worse disease-free survival (DFS) than patients with low KDM5B expression. **b** Patients with high KDM5B expression had a worse overall survival (OS) than patients with low KDM5B expression.100 HCC samples were divided into KDM5B high expression group (*n* = 50) and low expression group (*n* = 50), median was used as cut off

To investigate the clinical impact of elevated KDM5B expression in HCC, we assessed the association between KDM5B mRNA levels and clinicopathologic parameters in additional 100 patients with HCC. All HCC samples were divided into KDM5B high expression group (*n* = 50) and low expression group (*n* = 50), median was used as cut-off value. Significant correlations were found between KDM5B expression and tumor size, TNM stage, and Edmondson grade, suggesting that KDM5B might have a stimulatory role in the progression of HCC (Table 1). Moreover, Kaplan-Meier survival analysis was used to determine whether the expression of KDM5B was associated with disease-free survival (DFS) and overall survival (OS) of the HCC patients. The results showed that patients with high expression of KDM5B had a worse DFS than those with low KDM5B expression (*P* = 0.0005) (Fig. 2a). Likewise, a statistically significant association between high KDM5B expression and short OS was also demonstrated in HCC patients (*P* = 0.0006) (Fig. 2b). These results collectively implied that up-regulation of KDM5B can predict poor survival of HCC.

**Table 1:** Correlation between KDM5B expression and clinicopathological features in HCC

Clinicopathological features	n	KDM5B		
		High expression (≥Median)	Low expression (<Median)	*P*-value
Gender				
Male	65	34	31	0.6753
Female	35	16	19	
Age (years)				
≥ 50	60	32	28	0.5406
< 50	40	18	22	
Tumor size (cm)				
≥ 5	55	36	19	0.0012**
< 5	45	14	31	
Tumor number				
Solitary	62	33	29	0.5368
Multiple	38	17	21	
HBsAg				
Positive	76	40	36	0.4829
Negative	24	10	14	
HCV				
Positive	11	8	3	0.1997
Negative	89	42	47	
Cirrhosis				
Positive	82	43	39	0.4356
Negative	18	7	11	
ALT (U/L)				
≥ 45	46	26	20	0.3158
< 45	54	24	30	
AFP (ng/ml)				
≥ 20	57	31	26	0.4193
< 20	43	19	24	
TNM stage				
I + II	56	21	35	0.0085**
III + IV	44	29	15	
Edmondson grade				
I + II	59	23	36	0.0142*
III + IV	41	27	14	

The median expression level of KDM5B was used as the cut off

*P < 0.05

**P < 0.01 between the two groups

### KDM5B knockdown inhibits cell viability and colony formation

**Fig. 3:**
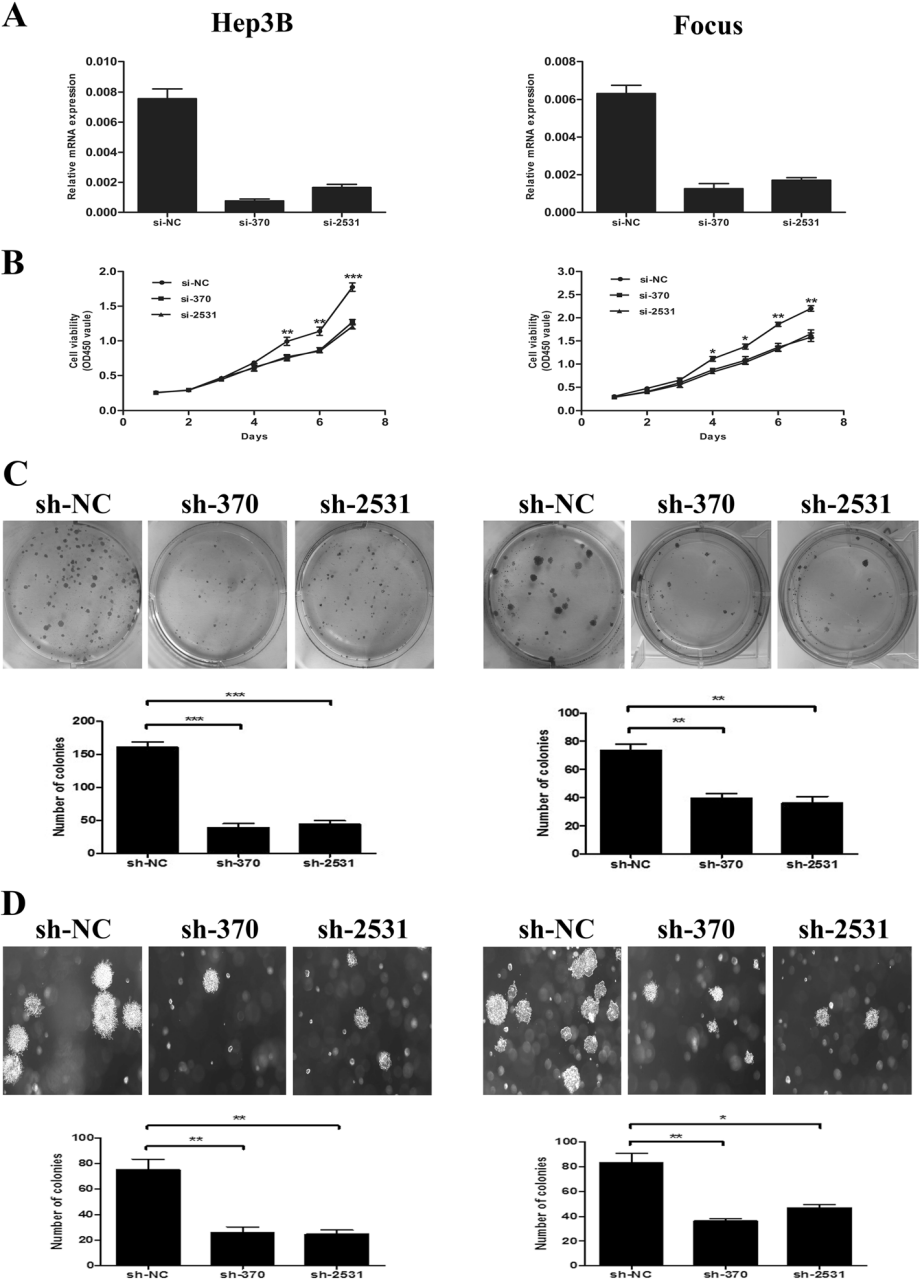
KDM5B knockdown inhibits HCC cellular proliferation and colony formation in vitro. **a** and **b** Knockdown of endogenous KDM5B suppresses the proliferation of Hep-3B and Focus cell lines. The silencing efficiency of two siRNA (si-370, si-2531) against endogenous KDM5B was evaluated by real-time PCR, where si-NC was used as a negative control. **c** KDM5B RNAi suppressed colony formation in Hep-3B and Focus cells, as shown by representative plates of cells transfected with the KDM5B shRNA constructs and shRNA-NC control. The histograms represent mean with standard deviation of colonies from triplicate tests. **d** KDM5B RNAi limited colony formation of Hep-3B and Focus cells in soft agar. The histograms represent mean with standard deviation of colonies from triplicate tests. Here all experiments were performed independently 3 times. A t test was used to evaluate the statistical significance of these experiments, as compared to the control. *, *P* < 0.05; **, *P* < 0.01; ***, *P* < 0.001. Original magnification, ×40

To determine whether KDM5B is necessary for the proliferation of HCC cells, we used chemically synthesised siRNAs and constructed the corresponding shRNA plasmids to knockdown endogenous KDM5B in 2 HCC cell lines (Hep3B and Focus) with relatively high KDM5B level. The efficient inhibition of KDM5B expression in siRNA-treated cells was verified using quantitative real-time PCR (Fig. 3a). As expected, we observed significant growth suppression of HCC cell lines treatd with siRNAs in comparison with the si-NC-transfected cells (Fig. 3b). Moreover, KDM5B depletion by shRNAs substantially inhibited the colony formation of KDM5B-overexpressing HCC cell lines compared to the control shRNA-NC-infected cells (Fig. 3c). Furthermore, down-regulation of kdm5B decreased the anchorage-independent growth of these HCC cell lines in soft agar and significantly reduced the number of larger colonies compared to the cells transfected with the negative control shRNAs (Fig. 3d). These collective data indicated that endogenous expression of KDM5B is essential for maintaining cell proliferation and colony formation in HCC cells and can function as an oncoprotein in HCC.

### KDM5B knockdown suppresses tumorigenicity and reduces tumor burden

**Fig. 4:**
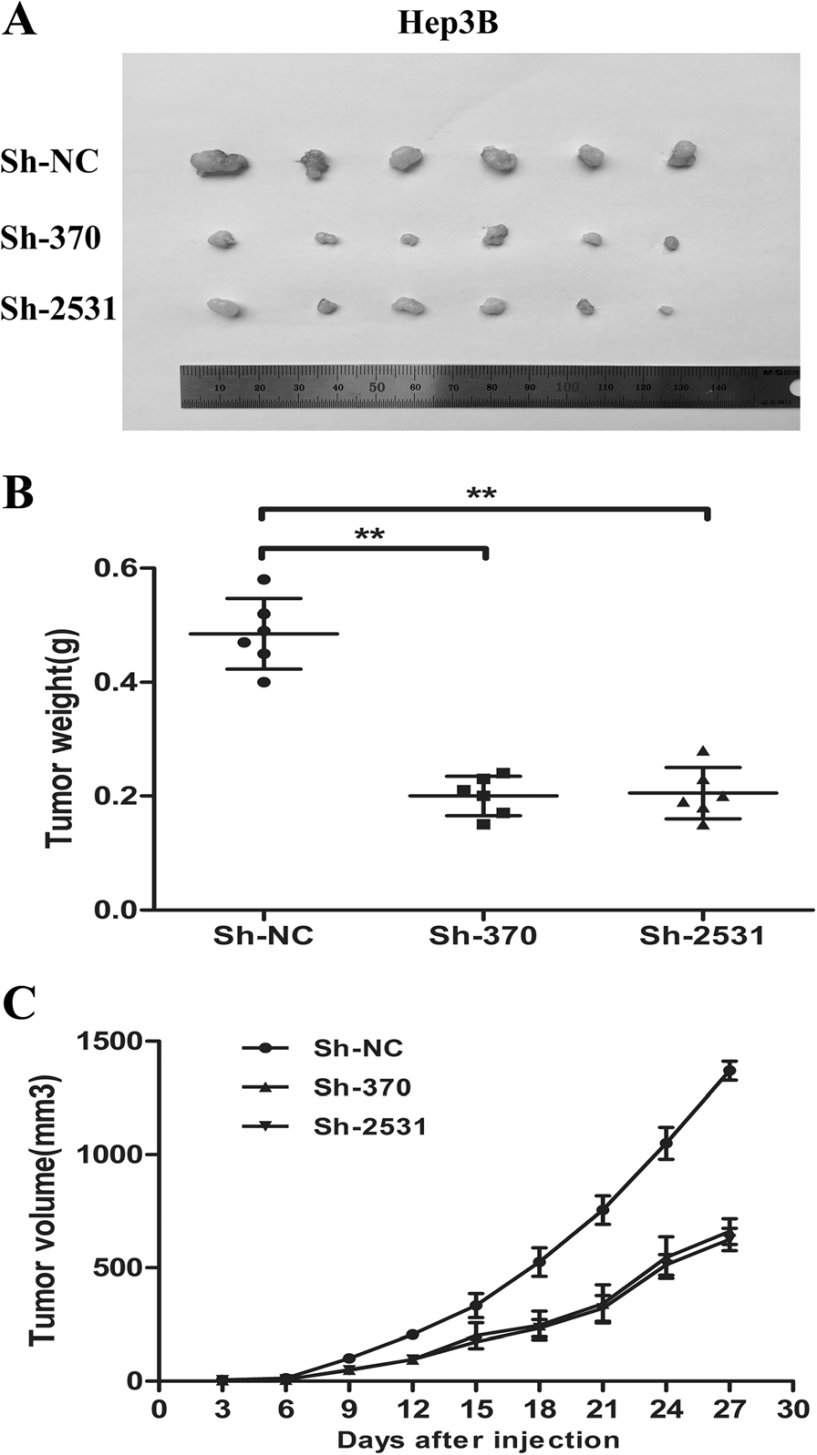
KDM5B knockdown reduces the tumor burden in vivo. **a**-**c** Both pSUPER-sh370 and pSUPER-sh2531 knockdown of KDM5B suppressed the tumorigenicity of Hep-3B cells in nude mice (*n* = 6), as compared with the negative control pSUPER-shNC. Tumors were weighed (**b**) and tumor volume (**c**) was evaluated every 3 days. The data were presented as the mean ± SD (*n* = 3), ** *P* < 0.01

To further determine the effect of KDM5B knockdown on tumorigenicity in vivo, we performed a subcutaneous xenograft tumor model in nude mice. Hep-3B cells were transfected with pSUPER-shRNA plasmids, then these cells were harvested and injected into the flanks of nude mice. The diameters of the tumors were measured every 3 days. As expected, pSUPER-sh370 and pSUPER-sh2531 were able to significantly suppress tumorigenicity, resulting in obvious reductions in tumor weight and volume compared to the negative controls pSUPER-shNC (Fig. 4a-c). All these results verified that KDM5B knockdown inhibited HCC cell xenograft formation and growth in vivo.

### KDM5B knockdown arrests the cell cycle at G1/S phase in HCC cells

**Fig. 5:**
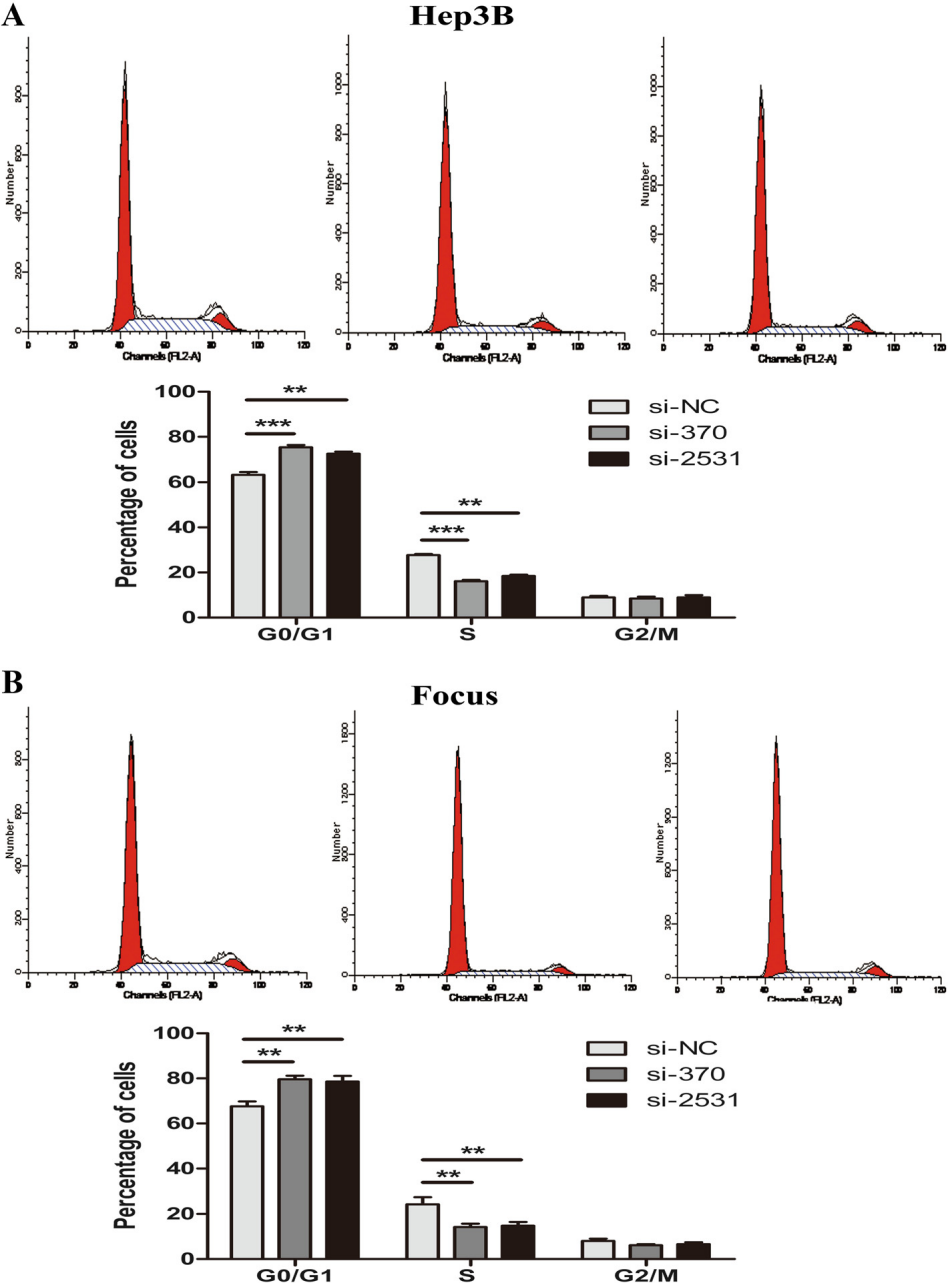
KDM5B knockdown causes cell cycle arrest in G0/G1 phase. **a** and **b** The cell cycle analysis was performed after Hep-3B cells or Focus cells were infected with siRNAs. Representative cell cycle distributions were shown and the histogram columns represent the average percentages of G0/G1, S and G2/M phases. The data were presented as the mean ± SD (*n* = 3), NS, not significant, **P < 0.01, and ***P < 0.001

To further assess the mechanism of growth suppression induced by KDM5B knockdown, we performed a flow cytometry to analyze the cell cycle status of cancer cells of cells with or without KDM5B depletion. The results showed that the fraction of HCC cells at the G1 phase was significantly higher in cells treated with siKDM5B than in those treated with control siRNAs, while the proportion in S phase was remarkably decreased (Fig. 5a and b). To investigate whether knock-down of KDM5B suppressed proliferation in HCC cells through apoptosis and senescence, we performed FACS assays and senescence-associated (SA) β-galactosidase (SA-β-gal) analysis, respectively. However, no apparent difference was observed in apoptotic cells (Additional file 1: Figure S1A) and senescent cells (Additional file 1: Figure S1B) between Hep-3B cells with or without KDM5B knockdown. Taken together, these data demonstrated that KDM5B affects HCC cell proliferation through cell cycle arrest, other than inducing cell apoptosis or senescence.

### KDM5B negatively regulates the expression of the p15 and p27

**Fig. 6:**
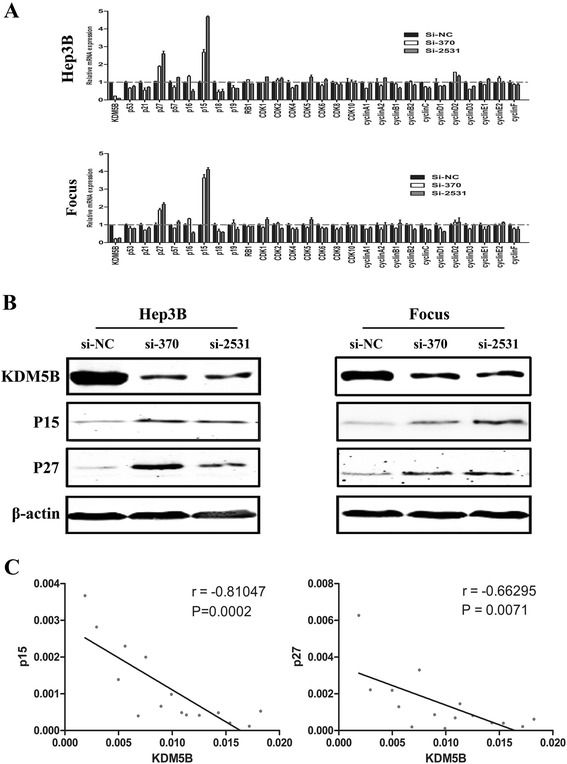
KDM5B depletion-mediated induction of CDKI p15 and p27. **a** KDM5B depletion obviously increased the mRNA expression of p15 and p27 in Hep3B and Focus cell line. **b** KDM5B depletion notably increased the protein expression of p15 and p27 in Hep3B and Focus cell line. **c** Pearson correlation analysis demonstrated the negative correlation between p15, p27 and KDM5B

The regulation of G1-S phase transition is governed by cyclins, cyclin-dependent kinases (CDKs), and cyclin-dependent kinase inhibitors (CDKIs). So we detected whether the expression of these genes was altered by KDM5B knockdown in HCC cells using quantitative real-time PCR. Interestingly, KDM5B depletion notably increased the mRNA expression of p15 and p27 in the Hep3B cell line. In contrast to p15 and p27, expression of other genes such as p14 and p16 were inconsistently or weakly affected by KDM5B knockdown. Similar results were obtained in the Focus cell line, indicating that KDM5B regulates p15 and p27 mRNA expression in multiple HCC cell lines (Fig. 6a). In addition, western blot results demonstrated that the protein levels of p15 and p27 were also increased with KDM5B silencing in Hep3B and Focus cells (Fig. 6b). We further examined whether p15 and p27 mRNA levels were in relation to KDM5B expression in 15 HCC cell lines by the Pearson correlation analysis. Results showed that CDKI expression was negatively correlated with KDM5B expression in the examined HCC cell lines (Fig. 6c). These data suggested that p15 and p27 expression can be negatively regulated by KDM5B in HCC cells.

### Loss of the KDM5B occupancy was coupled with elevated H3-K4 trimethylation on the p15 and p27 promoters in KDM5B-depleted HCC cells

**Fig. 7:**
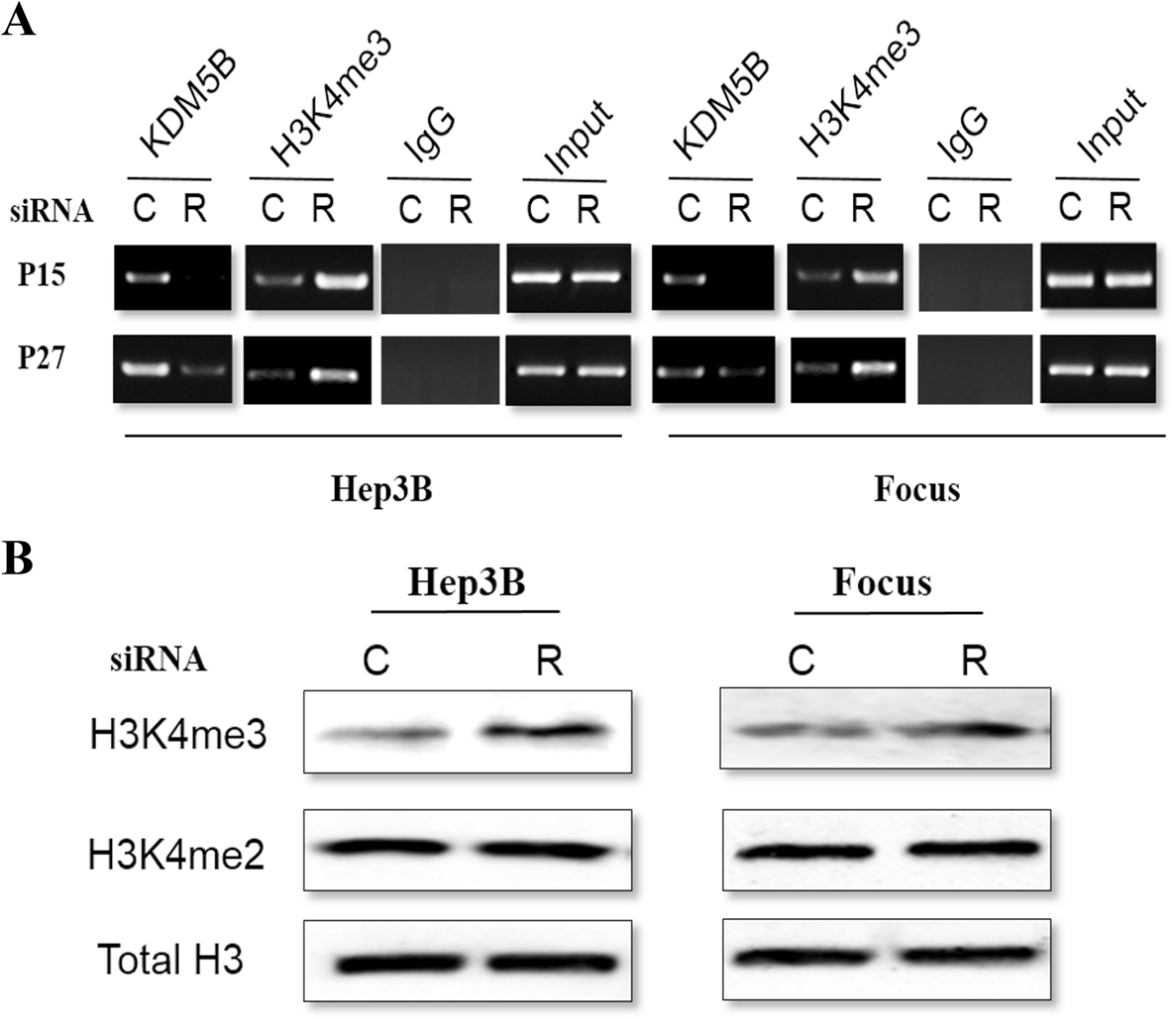
Regulation of the p15 and p27 promoter occupancy on their promoters region by KDM5B. **a** and **b** The KDM5B occupancy and trimethylation status of histone H3 (H3K4Me3) at the promoters of p15 and p27 genes. C and R: Control siRNA and KDM5B siRNA, respectively. ChIP was used to precipitate KDM5B and H3K4Me3-bound DNA followed by PCR amplification with the specific primers spanning p15 and p27 promoters. Input: 2 % of total chromatin materials were PCR amplified with the same primers

Since p15 and p27 expression were regulated by KDM5B at both mRNA and protein levels, we speculated whether these 2 genes are direct targets of KDM5B. Chromatin immunoprecipitation assay was thus performed to determine an association of KDM5B with p15 and p27 promoters. In the cells treated with control siRNA, KDM5B occupancy on the promoter region of the 2 genes was readily detectable. In contrast, knocking down KDM5B abolished its association with these promoter sequences (Fig. 7a and b). Moreover, KDM5B depletion led to significantly enhanced H3-K4 trimethylation at the proximal promoter region of p15 and p27 genes (Fig. 7a and b). These consistent results suggested that p15 and p27 are candidate direct target genes negatively regulated by KDM5B in HCC cells.

### KDM5B regulates HCC cell growth in a p15- and p27- dependent manner

**Fig. 8:**
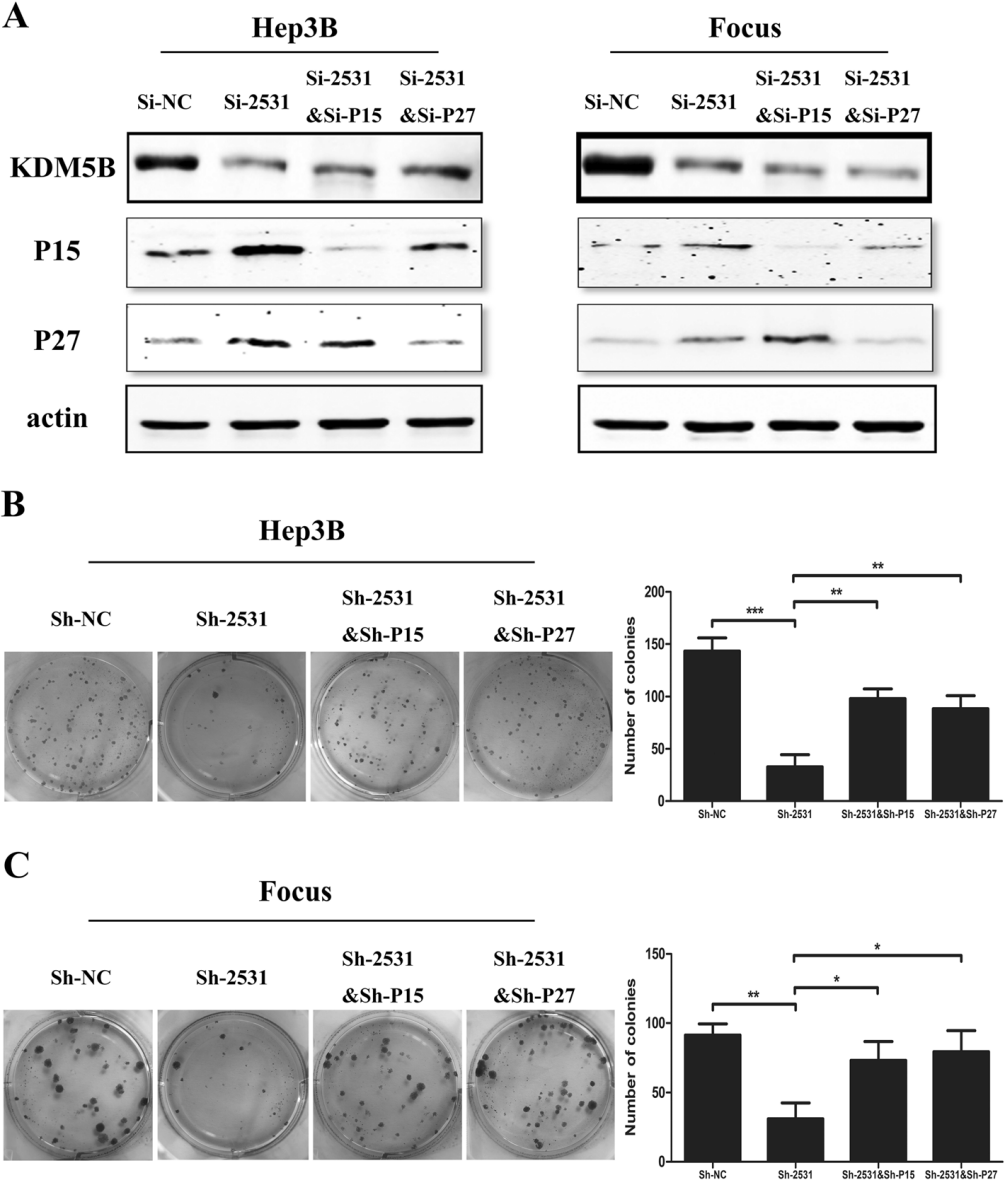
Partial recovery of clonogenic potential in KDM5B-depleted HCC cells by inhibition of p15 and p27 induction. **a** Western blot analyses of KDM5B, p15 and p27 protein expression in Hep-3B and Focus cells treated with si-NC, si-2531, co-transfection of si-2531 and si-p15 and co-transfection of si-2531 and si-p27. **b** and **c** The restoration of clone formation in KDM5B-depleted Hep-3B and Focus cells by abolishment of p15 and p27 induction. *, *P* < 0.05 and **, *P* < 0.01, respectively

To determine whether p15 and p27 are key mediators of KDM5B’s function in cellular proliferation, we assessed the colony formation when both KDM5B and p15 or p27 were simultaneously silenced via RNAi (Fig. 8a). Interestingly, RNAi against p15 or p27 could largely rescue the inhibitory effect of KDM5B knockdown on colony formation in Hep3B (Fig. 8b) and Focus (Fig. 8c) cells. These results indicated that the inhibition of cellular proliferation caused by KDM5B knockdown are dependent largely on the up-regulation of p15 and p27.

## Discussion

Hepatocellular carcinoma (HCC) is a worldwide malignancy with a high mobility and mortality, especially in China. The long-term survival rate for HCC remains poor despite advanced therapeutic modalities and strategies such as surgery, chemotherapy and radiotherapy. Therefore, better understanding of the pathogenesis of HCC and exploring novel therapeutic approaches against drug targets are urgently needed.

Emerging evidence has indicated that a number of histone methylation modifiers are dysregulated in tumors and are critical for oncogenic phenotypes [35–38]. Jumonji C (JmjC) domain containing protein has been identified to possess histone demethylase activity and play important roles in cancer ignition and progression. KDM5B, a member of the KDM5 family of proteins, has recently been shown to possess H3-K4 demethylase activity and exhibit oncogenic activities in several human cancers. However, whether and how KDM5B is involved in HCC progression is still unknown. Here, we provided evidence that KDM5B was frequently up-regulated in HCC tissues and that its expression in HCC cells is required to repress the transcription of multiple CDKIs and to maintain sustained proliferation of cancer cells. These immortal malignant cells undergo cell cycle arrest and lose clonogenic potentials when KDM5B is knocked down. Therefore, we speculated that the aberrant expression of KDM5B might be an important epigenetic factor involved in the pathogenesis of HCC.

The effect of KDM5B inhibition on cell proliferation is not cell type or context-specific, since we observed similar phenotypic changes in two different HCC cell lines (Hep3B and Focus) upon KDM5B knockdown. Analysis of cell cycle distribution revealed that KDM5B depletion led to an obvious arrest at G0/G1 phase. Moreover, results of FACS assays and senescence-associated (SA) β-galactosidase (SA-β-gal) analysis excluded the possibility that KDM5B knockdown affected HCC cell proliferation through apoptosis or senescence. Therefore, we decided to lay emphasis on some molecules involved in the regulation of G1-S phase transition. P15, a member of the INK4 family of cyclin-dependent kinase inhibitors, is capable of inducing cell cycle arrest in G1 phase and has been identified as a tumor suppressor, as well as the other two INK4 family members p16 and p14 [39, 40]. P27 is also a cyclin-dependent kinase inhibitor and can block the cell cycle at G0/G1 phase upon differentiation signals or cellular insult. The degradation of p27 protein is required for the cellular transition from quiescence to the proliferative state [41–43]. H3-K4 methylation and demethylation are prevalent marks associated with transcription activation and repression. KDM5B, which is a histone demethylase specific for di- and trimethylated H3-K4, is believed to act as a transcriptional repressor partly through inhibiting H3-K4 methylation at its target promoters. Actually, KDM5B has been implicated in silencing tumor suppressor genes such as BRCA, p21 and TIEG1 through its demethylase activity [25, 28, 44]. According to this model, our findings provide evidence that p15 and p27 are all the direct targets of KDM5B in HCC. First, our current results demonstrated that depletion of KDM5B notably up-regulated the mRNA and protein expression of p15 and p27. In addition, KDM5B was associated with the proximal promoter sequences of these 2 genes. When knocked down, KDM5B occupancy on these promoters was lost, which was coupled with highly elevated H3-K4 trimethylation locally. Moreover, loss of p15 and p27 function could largely rescue the anti-proliferation effect of KDM5B depletion on HCC cells, although the recovery was not 100 % efficient compared with control cells. Given the fact that these 2 proteins are tumor suppressors by negatively regulating cell cycle progression, we conclude that up-regulation of KDM5B may act as a proto-oncogene which functions at least partially by repressing tumor suppressors through its demethylase activity.

## Conclusions

In conclusion, our results suggest that KDM5B is overexpressed in the vast majority of HCC specimens and that its depletion leads to highly impaired clonogenesis and cell cycle arrest through up-regulation of p15 and p27. Therefore, KDM5B could be a potentially therapeutic target, which provides a rationale for the development of histone demethylase inhibitors as a strategy against HCC. 

## Additional file

Additional file 1: Figure S1. Knockdown of KDM5B has no obvious effect on apoptosis and senescence of HCC cells. (A) FACS assays was performed to detect the difference in apoptotic cells between Hep-3B cells with or without KDM5B knockdown. (B) Senescence-associated (SA) β-galactosidase (SA-β-gal) analysis was performed to detect the difference in apoptotic cells between Hep-3B cells with or without KDM5B knockdown. The data were presented as the mean ± SD (*n* = 3), NS, not significant, ***P* < 0.01, and *** *P* < 0.001. (DOCX 656 kb)

## Abbreviations

HCC: hepatocellular carcinoma
KDM5B: lysine (K)-specific demethylase 5B
shRNA: short hairpin RNA
siRNA: small interference RNA
ChIP: chromatin immunoprecipitation
GEO: gene expression omnibus
CDKN1B: cyclin-dependent kinase inhibitor 1B (P27)
CDKN2B: cyclin-dependent kinase inhibitor 2B (P15)
