# Supplementary material for: Depletion of histone demethylase KDM5B inhibits cell proliferation of hepatocellular carcinoma by regulation of cell cycle checkpoint proteins p15 and p27
Source: J Exp Clin Cancer Res. 2016 Feb 25;35:37. doi: 10.1186/s13046-016-0311-5 (PMC4766611; doi:10.1186/s13046-016-0311-5)
Supplement: Additional file 1: Figure S1. — Knockdown of KDM5B has no obvious effect on apoptosis and senescence of HCC cells. (A) FACS assays was performed to detect the difference in apoptotic cells between Hep-3B cells with or without KDM5B knockdown. (B) Senescence-associated (SA) β-galactosidase (SA-β-gal) analysis was performed to detect the difference in apoptotic cells between Hep-3B cells with or without KDM5B knockdown. The data were presented as the mean ± SD (n = 3), NS, not significant, **P < 0.01, and *** P < 0.001. (DOCX 656 kb) [file 13046_2016_311_MOESM1_ESM.docx]

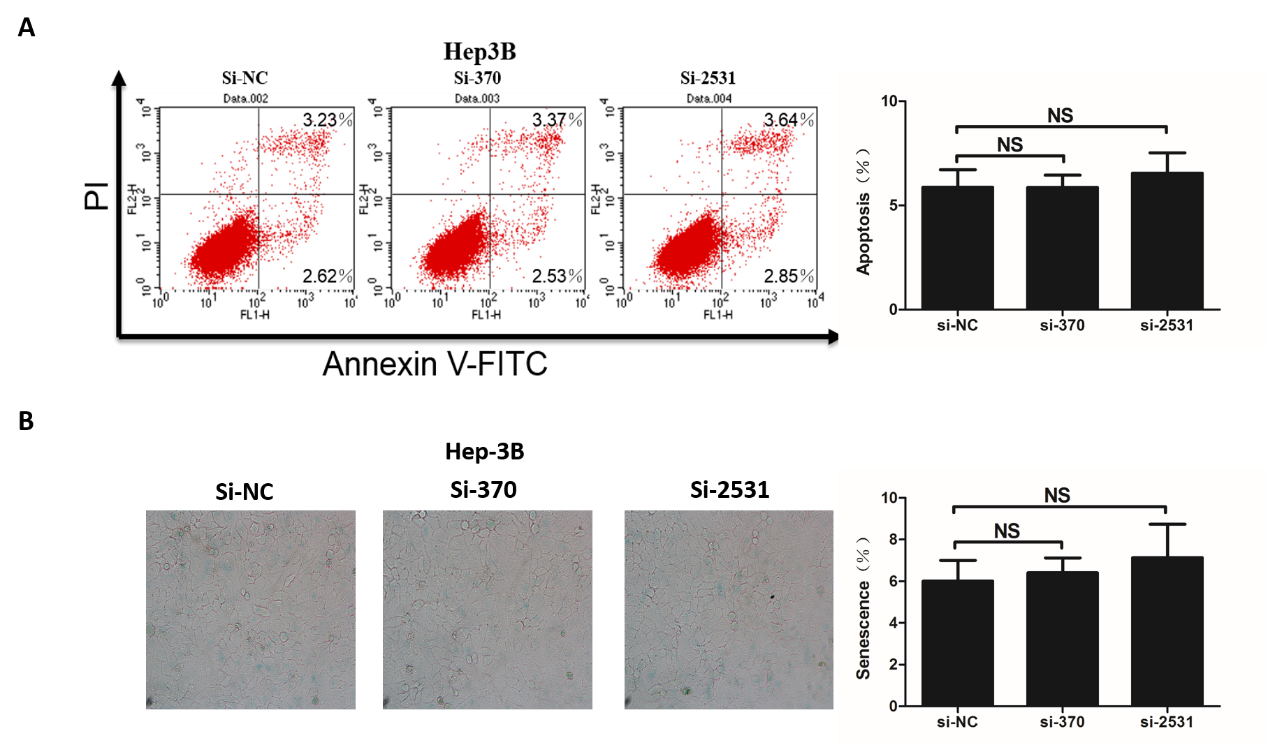


Supplementary Fig. 1. Knockdown of KDM5B has no obvious effect on apoptosis and senescence of HCC cells. (A) FACS assays was performed to detect the difference in apoptotic cells between Hep-3B cells with or without KDM5B knockdown. (B) Senescence-associated (SA) β-galactosidase (SA-β-gal) analysis was performed to detect the difference in apoptotic cells between Hep-3B cells with or without KDM5B knockdown. The data were presented as the mean ± SD (n=3), NS, not significant, **P < 0.01, and *** P < 0.001.
